# Obesity and Multimorbidity in the USA: National Health and Nutrition Examination Surveys 2005-2014

**DOI:** 10.1093/geroni/igab046.2348

**Published:** 2021-12-17

**Authors:** David Lynch, Curtis Petersen, Hillary Spangler, Anna Kahkoska, John Batsis

**Affiliations:** 1 University of North Carolina, Chapel Hill, North Carolina, United States; 2 Dartmouth College, Lebanon, New Hampshire, United States; 3 UNC Hospitals, Chapel Hill, North Carolina, United States; 4 University of North Carolina at Chapel Hill, University of North Carolina at Chapel Hill/Chapel Hill, North Carolina, United States; 5 University of North Carolina at Chapel Hill, Chapel Hill, North Carolina, United States

## Abstract

Declining mortality rates and an aging population have contributed to increasing rates of multimorbidity (≥2 chronic conditions) in the United States. Obesity is an important risk factor for the development of chronic diseases. We evaluated the association between obesity and multimorbidity, and how the prevalence of concomitant obesity has changed over time. We used data from 8,883 individuals aged ≥60 years with data on body mass index (BMI) and self-reported comorbidities from the National Health and Nutrition Examination Surveys 2005-2014. Logistic regression was used to quantify the association between BMI categories (<18.5, 18.5-24.9, 25-29.9, ≥30 kg/m2) and multimorbidity (yes/no). Change in proportions of obesity coexisting with multimorbidity by year was tested through linear regression. All analysis used NHANES survey design and weighting to be representative of the US population. The overall proportion of individuals with concomitant multimorbidity and obesity was 75%. As compared to a normal BMI (18.5-24.9 kg/m2), older adults with obesity (BMI ≥30 kg/m2) had higher odds of multimorbidity (OR 1.78, 95% CI 1.49,2.12). Persons with obesity had higher odds of decline in physical (1.41 [1.06,1.88]), basic (1.56 [1.13,2.15]), and instrumental activities of daily living (OR 1.58 [1.03,2.40]). The proportion of individuals with obesity and multimorbidity increased over time, but did not reach significance (β = 0.008, p=0.051). These results emphasize the role of obesity as a contributing factor to the burden of multimorbidity among older adults and underscore the importance of identifying and addressing obesity and multimorbidity via interventions to decrease obesity prevalence.

